# Targeted resequencing of HIV variants by microarray thermodynamics

**DOI:** 10.1093/nar/gkt682

**Published:** 2013-08-08

**Authors:** Wahyu W. Hadiwikarta, Bieke Van Dorst, Karen Hollanders, Lieven Stuyver, Enrico Carlon, Jef Hooyberghs

**Affiliations:** ^1^Flemish Institute for Technological Research, VITO, Boeretang 200, B-2400 Mol, Belgium, ^2^Institute for Theoretical Physics, KULeuven, Celestijnenlaan 200D, B-3001 Leuven, Belgium, ^3^Janssen Diagnostics bvba, Turnhoutseweg 30, B-2340 Beerse, Belgium and ^4^Theoretical Physics, Hasselt University, Campus Diepenbeek, Agoralaan - Building D, B-3590, Diepenbeek, Belgium

## Abstract

Within a single infected individual, a virus population can have a high genomic variability. In the case of HIV, several mutations can be present even in a small genomic window of 20–30 nucleotides. For diagnostics purposes, it is often needed to resequence genomic subsets where crucial mutations are known to occur. In this article, we address this issue using DNA microarrays and inputs from hybridization thermodynamics. Hybridization signals from multiple probes are analysed, including strong signals from perfectly matching (PM) probes and a large amount of weaker cross-hybridization signals from mismatching (MM) probes. The latter are crucial in the data analysis. Seven coded clinical samples (HIV-1) are analyzed, and the microarray results are in full concordance with Sanger sequencing data. Moreover, the thermodynamic analysis of microarray signals resolves inherent ambiguities in Sanger data of mixed samples and provides additional clinically relevant information. These results show the reliability and added value of DNA microarrays for point-of-care diagnostic purposes.

## INTRODUCTION

In human genetic research, targeted resequencing of genomic nucleic acid is applied extensively in population studies to search for associations between sequence variants and diseases. The technique is also applied in diagnostic or prognostic tests. In this context, one is often confronted with samples of mixed sequence variants, some possibly present in minority: e.g. biopsies from cancer tissue usually contain a mixture of cancerous and non-cancerous cells. Therefore, one needs to distinguish the presence of a specific mutation, possibly in low abundance (minority), in a majority of ‘wild-type’ sequences. Several techniques are in use to resolve the sequence composition in mixed sequence samples, like allele-specific PCR (1), melting curve analysis or sequencing (2).

In this article, we focus on the case of HIV-1/AIDS. To reduce the morbidity and mortality world-wide, there is a high need for simple point-of-care genotyping tests to screen for key resistance mutations. However, the development of a simple genotyping test to screen for key resistance mutations is a technical challenge, due to high genetic variability of HIV-1 (3). The variability is caused by the error-prone reverse transcriptase enzyme, which will introduce mutations during each replication cycle, combined with a short replication time. Within one single patient, different, but closely related, non-identical viral genomes can be present. This high variability makes the design of primers and probes for a simple genotyping assay difficult. A variety of high throughput genotyping assay for antiretroviral resistance testing are available in the market, which allow physicians to determine drug-resistance profiles (4–6). These genotyping assays are using capillary electrophoresis platforms that provide integrated systems for nucleotide sequence-based analysis and interpretation for drug-resistance mutations in the HIV-1 reverse transcriptase and protease. The development of these assays greatly advanced clinical care for HIV-1 patients, by allowing personalized disease management, using the most appropriate drugs and drug combinations available at any given point (6). These assays are requiring high-tech equipment and are performed by highly trained laboratory personnel limiting their practical use as point-of-care test.

In this article, a new method based on microarrays hybridization will be introduced to perform targeted resequencing on nucleic acid samples. The idea to use hybridization for mutation detection is not new (7,8), and it has often been compared with other techniques (9,10). An advantage of hybridization is its simplicity and the possibility of miniaturization for point-of-care tests. An often reported disadvantage is its specificity: the possibility of cross-hybridization of not-perfectly matching sequences to a probe sequence complicates the data analysis. The situation complicates even further when the original sample contains two or more variants of a given sequence. As usually cross-hybridization is viewed as a limiting factor, efforts are often aimed at avoiding it, e.g. by introducing chemical agents in the nucleic acid probes (11,12). In this article, we show that if the probe-target affinities for cross-hybridization are quantified accurately, the measurements from multiple probes, which typically are not perfectly matching to the sample sequences, can be turned into a powerful targeted resequencing method. The analysis relies on estimates of the hybridization free energies of mismatching duplexes (cross-hybridization signals). The data are then checked against the isotherm expected from equilibrium thermodynamics (13–15).

## MATERIALS AND METHODS

### HIV samples

HIV-1 virus stocks were selected from the Janssen Diagnostics repository database, based on their known mutation profile in the region of codon 179 to codon 186 of the Reverse Transcriptase (RT) gene. This region was selected to cover key resistance mutations at position 179, 181 and 184. A mutation at position 179 or 181 causes resistance against non-nucleoside RT inhibitors (NNRTIs) (16–20), while nucleoside RT inhibitors (NRTIs) are selecting for a mutation at position 184 (21,22). An informed consent for research purposes is available for the HIV samples used.

### Experimental protocol

The viral RNA extraction of virus stocks was carried out on an EasyMAG (bioMérieux, Boxtel, The Netherlands) according to the guidelines of the manufacturer, starting with 256 µl input material for plasma samples and 100 µl input material for virus stocks, both were eluted in 60 µl. A One-Step RT-PCR amplification (One-Step Superscript III HiFi, Invitrogen, CA, USA) was used to generate a 2.3-kb HIV-1 fragment (containing the gag-protease-reverse transcriptase (GPRT) region) using the 3-RT (5′-CATTGCTCTCCAATTACTGTGATATTTCTCATG-3′) and 5-OUT (5′-GCCCCTAGGAAAAAGGGCTGTTGG-3′) primers. RNA input was 10 µl in a final volume of 35 µl. The complete amplification procedure was published by (23). The 2.3-kb HIV-1 outer fragment generated with the GPRT one-step PCR was used as template for the asymmetric amplification of the sequence around RT codon 184. Therefore, the HIV-1_Fw_184Cy3(5′-/Cy3/TAGAAAACAAAATCCAGAAATA-3′) and HIV-1_Rev_184 (5′-TGCCCTATTTCTAAGTCAGATCC-3′) primers were used, with the fluorescent labelled forward primer HIV-1_Fw_184Cy3 in excess to generate fluorescent labelled single-stranded DNA (ssDNA) fragments of 78 bp (containing RT codon 184). Forward primer HIV-1_Fw_184Cy3 and reversed primer HIV-1_Rev_184 were used at a concentration of 1 µM and 0.1 µM, respectively, with DNA input of 2 µl in a final volume of 100 µl. The microarray experiments were performed in an Agilent platform. Each experiment was performed, following the standard protocol discussed in (24). We considered hybridizing sequences of 25 nt. This is because in previous studies (13), sequences of this length were found to attain thermodynamic equilibrium after 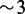
h of hybridization (in the experiments, the hybridization time is of 17 h to ensure that equilibrium was reached).

The raw microarray data were subjected to a primary quality control using the Agilent Feature Extraction Software (Version 10.7). For all arrays, the spot centroids in the four corners of the microarray have been located properly and consequently the grid was placed correctly. The QC values for homogeneity and those for checking the hybridization and washing steps, all fall within the good range according to Agilent guidelines. In the present application, we work with single color, hence there is no need of color normalization. In addition, the analysis in extended previous hybridization experiments (13,15) showed that the experimental data closely follow the thermodynamics models in the full range of measured experimental intensities without additional normalization requirements.

### Microarray design

Table 1 shows some sequences with high clinical frequency obtained with Sanger sequencing from a database provided by Janssen Diagnostics, of about 350 000 patients, on a region of 25 nt of the HIV-RT centred around codon 184. The sequence with the highest frequency occurs in only 25% of the patients. The other 75% of the cases contain mutations with respect to it. This makes the HIV an excellent test model to check the validity of the method presented in this article. Part of the samples in Table 1 consist of unique sequences. Other samples are mixed, i.e. HIV viruses with sequence differences within the 25-nt window considered coexist in a single patient. Further, the concept of unique and mixed sample has to be interpreted within the limited sensitivity of Sanger sequencing. This method can detect a mixture only if the relative abundance of the low abundance sequence is >20%. For instance, a mixed sequence sample with only 10% of low abundance sequence would be detected as unique sequence sample by the Sanger method (2).

**Table 1.** Nucleotide sequences (from codon 179 to codon 186, written in 5′ to 3′ orientation) for different variants of the HIV-RT gene, as obtained from the analysis of 350 000 patients

Rank	Type	Sequence	Relative clinical
		…180…182…184…186.	Frequency
1	Unique	GTTATCTATCAATACATGGATGATT	1.000
2	Unique	GTTATCTATCAATAC**G**TGGATGATT	0.411
3	Unique	GTTATCTATCAATACATGGATGA**C**T	0.124
4	Unique	GT**C**ATCTATCAATACATGGATGATT	0.084
5	Unique	GTTATCT**G**TCAATACATGGATGATT	0.075
		⋮	
9	Unique	GTTATCTATCAATAC**G**TGGATGA**C**T	0.056
		⋮	
15	Mixed	GTTATCTATCAATAC**R**TGGATGATT	0.037
		⋮	
22	Unique	GT**C**ATCTATCAATA**T**ATGGATGA**C**T	0.027
		⋮	
143	Unique	GTTATCTATCAATACATGGATGA**CC**	0.002
		⋮	

The database, provided by Janssen Diagnostics, is obtained from Sanger sequencing, and only some selected sequences are shown here. The ranking follows the relative clinical frequency. Numbers above the first ranked sequence indicate the codons position. Nucleotides differing from those of the most frequent sequence are shown in bold. The Sanger sequencing method yields either *unique* sequences, i.e. with no ambiguities, or *mixed* sequences. In the Table, the mixed sequence with the highest clinical frequency is ranked no. 15. We use here the standard notation for degenerate bases (25); therefore, R means a purine (A or G). Note that this sequence is a mix between sequences ranked no. 1 and no. 2 in the Table. The sequence ranked no. 143 is the 100th unique sequence from the database and so is the last in the PM set.

To define a manageable and relevant diagnostic problem, each unique sample from the database was selected and ranked in decreasing order of clinical frequency. The goal was to diagnose the top 100 unique sequences and mixtures of two sequences thereof. This corresponds to the coverage of 82% of the whole database. The sequence no.143 shown in Table 1 is the 100th unique sequence relative to the most frequent one. This sequence has two mismatches with respect to the most frequent one.

The 15k custom Agilent array used in the experiment contains 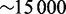 spots. The design of the probe sequences on the array was started from the 100 probes that are perfectly matching to the 100 unique sequences considered in the test (referred as the perfect match (PM) set in the design). The data analysis of the method discussed in this article is not solely based on the signal of perfectly matching spot, but also on cross-hybridizing signals. Previous experiments (15,25) showed that signals measured from spots with one or two mismatches with respect to the target sequence are still above the lower limit of detection. Therefore, probes with one or two mismatches with respect to those in the PM set were included in the microarray. The final design contains 2139 different probes replicated seven times to fill all the available spots of the 15k microarray.

### Thermodynamic assessment

In the standard approach of hybridization-based targeted resequencing, the microarray contains probes that are perfectly matching to all possible variants of target sequences expected to be present in the biological sample. The brightest of all spots should indicate the sequence which is present in solution. Our approach is based on the analysis of the intensities of the brightest and also of many ‘dimmer’ spots which are expected to carry one or two mismatches with respect to the target (actually the large majority of signals comes from hybridizations to mismatching sequences). If a unique target sequence is present in solution, the fluorescence intensity *I* from different spots will be correlated according to equilibrium thermodynamics as
(1)
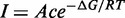

where *A* is a proportionality factor, *c* the target concentration in solution, 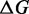 the hybridization free energy as a sequence-dependent measure of the affinity between probe-target sequences, *R* the gas constant, and *T* the temperature. For convenience in the rest of the article, hybridization free energies are shifted by the corresponding perfect match value. This amounts to use 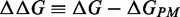; therefore, for a PM hybridization 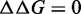. Note that as the free energies are shifted by a constant value, the same functional relationship as Equation (1), holds also for 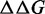. Equation (1) holds at sufficiently low concentrations, while at high concentrations saturation effects should be taken into account, as the intensity reaches a maximal value when all probes are hybridized [as predicted by the Langmuir model (24)]. These effects are not relevant in the range of concentrations investigated here. Equation (1) was thoroughly tested and validated in experiments on Agilent arrays using several different sequences (15,25).

Given the sequences of the target in solution and the surface-bound probes, the 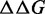 can be obtained from the nearest-neighbour model (26). In this model, the hybridization free energy is given by the sum of parameters that are dependent on pairs of neighbouring nucleotides. The computation of 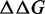 used in this article follows the same principles described in (15,24).

**Figure 1.**
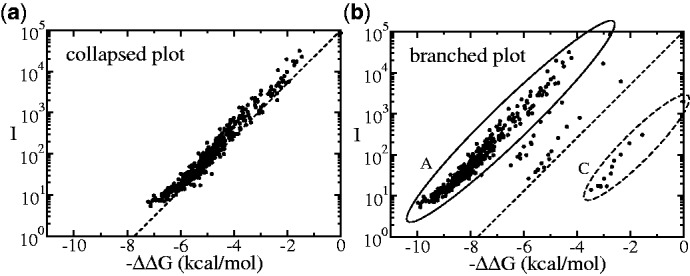
Two *I* versus 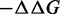 plots from the same set of experimental data for the hybridization of a synthetic oligomer target sequence 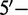
AAGGGCCACGGATTACTCGTAATAA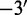 to a microarray containing a perfect match probe and many probes with one or two mismatches with respect to the target. The 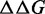 are calculated using the nearest neighbour model free energies by means that are described in (15,24), using two different hypotheses for the target sequence in solution. In (**a**) the hypothesis matches the actual target sequence and the data follows the Equation (1) that is shown as a dashed line. In (**b**) the hypothesis differs by one nucleotide with respect to the actual target sequence in solution. Four different branches appear in this plot. The origin of these branches is discussed in the text.

In a resequencing analysis, the target sequences are coming from a biological sample, and they are usually not known. One can, however, make a starting hypothesis about the sequence and compute the corresponding 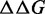. If the starting hypothesis agrees with the actual sequence in the sample, the measured intensities should be distributed according to Equation (1). Deviations from this law may have two causes: (i) The starting hypothesis is wrong; hence, the sequence in the sample is different from what originally assumed or (ii) the sample is a mixture, i.e. it contains the sequence of the original hypothesis together with other sequences. This concept is illustrated in Figure 1 that shows plots of *I* versus 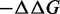 in log-linear scale for which Equation (1) becomes a straight line with slope 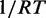 (shown as dashed line). The two plots refer to the same experimental data. In one case (Figure 1a), the hypothesis matches the actual sequence in the sample. The data accurately follow Equation (1) over four orders of magnitude in the intensity scale. In the other case (Figure 1b), the wrong hypothesis leads to an incorrect computation of the 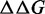 and deviations from the expected thermodynamic behaviour. Here, the data are distributed into four distinct branches. The origin of this branching will be discussed in detail in the next section, which presents an algorithm used to infer the sequence composition from the analysis of the plots of the measured fluorescence intensities *I* versus 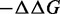. We refer these plots as 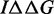-plots.

### Algorithm

**Figure 2.**
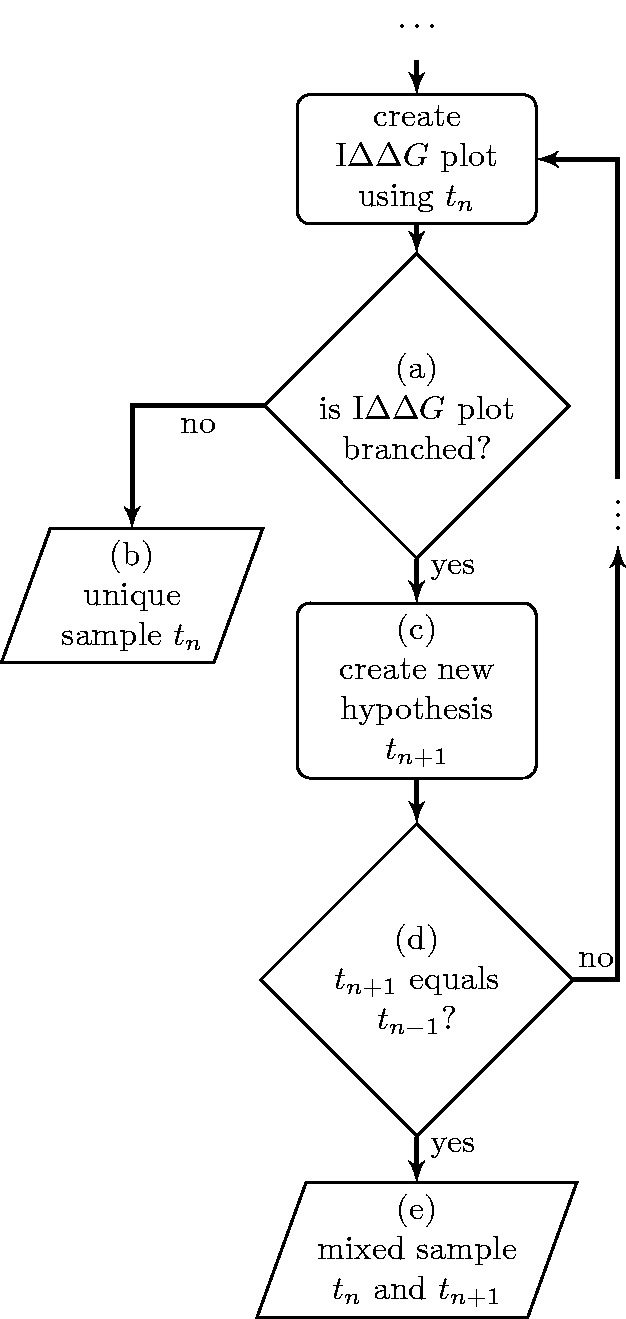
A flowchart showing the basics of the algorithm for targeted resequencing of HIV-RT. *t_n_* is the hypothesis for the target sequence generated at the *n*-th iteration. The outputs are either a unique sequence [block (b)] or a mixed sequence composed by two sequences [block (e)] depending on the nature of the 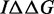 plots.

Figure 2 shows a flowchart of the algorithm used for the targeted resequencing. The algorithm consists of a central loop that generates iteratively sequences 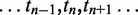 (where *n* is the iteration step), which are successive *in silico* hypotheses about the composition of the sample. The loop is repeated until convergence is reached. Two types of convergence are obtained. In some cases, after a certain number of iterations, the algorithm shows a collapsed 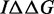 plot similar to that seen in Figure 1a. This is a signature of the presence of a unique sequence in the sample. In this case, the algorithm ends at the block (b) of the flowchart and returns the output *t_n_* as the sequence composition of the sample. In other cases, the algorithm converges to a two-cycle state i.e. 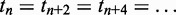 and 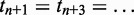, where 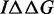 plots are always branched (similar to that seen in Figure 1b). This two-cycle state is a signature for a sample composed by a mixture of two sequences: *t_n_* and 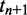. In this case, the algorithm ends at the block (e) and returns the aforementioned two sequences as output.

An important part for generating new *in silico* hypotheses is the decision block (a) of the flowchart. At this point, the algorithm checks if the 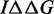 plot is collapsed or branched. In the latter case, a new *in silico* hypothesis 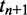 is generated. To understand how this is done, the origin of the branching has to be elucidated in some detail.

**Figure 3.**
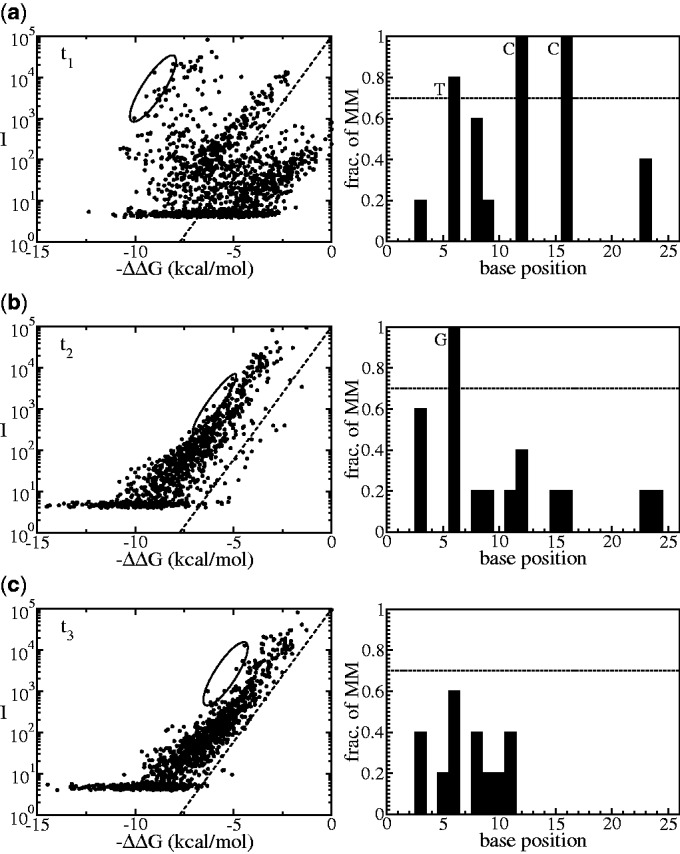
Analysis on sample no. 4; the first iteration tells that the hypothesis *t*_1_ is not correct, as the 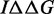 plot is branched (**a**). Analysis on the selected deviating points (encircled) reveals mismatching nucleotides per base positions that are commonly found in the selected probes. These nucleotides become the basis to generate a new hypothesis *t*_2_. In the second iteration with the new hypothesis *t*_2_, the 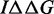 plot is apparently still branched (**b**); thus, the iteration continued by generating another new sequence *t*_3_. The 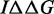 plot with hypothesis *t*_3_ turns out to be a collapsed plot; thus, the algorithm stops and conclude that the sample contains unique sequence (**c**).

The plot of Figure 1b is obtained by calculating 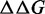 using a wrong *in silico* hypothesis: Table 2 shows the actual sequence in solution used in the experiment to produce Figure 1a and the hypothesis sequence made for the calculation of Figure 1b. They differ by a single nucleotide at base position 13 (in the *in silico* hypothesis this is a G, while the actual target contains a T). Consider now all probe sequences in the microarray with an A at this position. The actual hybridization is a Watson-Crick AT pairing, while the *in silico* hypothesis estimates the 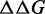 as these were AG mismatches. This leads to overestimating the 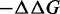. The data points corresponding to the probes with nucleotide A at base position 13, are encircled with a solid line in Figure 1b. Conversely, for the probes with a nucleotide C the 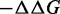 are underestimated. The latter are encircled with a dashed line in Figure 1b. Thus, the splitting into four branches is due to the wrong estimates of free energy for each probe in the position where actual target and *in silico* hypothesis differ. It is important to notice here that the probes in the different branches systematically differ from each other by specific nucleotides at specific locations. This systematic sequence deviation will be used to decide whether the 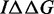 plot is branched or not (see right panes in Figures 3 and 5 of the examples in the next section). The correct hypothesis can readily be constructed by selecting out the top left branch, determining the systematic sequence deviation (nucleotide position and type) in this probe subset, and implementing this nucleotide change in the previous hypothesis. This is precisely how a new *in silico* hypothesis denoted by 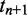 is generated in block (c) by the algorithm.

**Table 2.** Origin of branches appearing in the 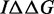 plot

Targets	Actual	Actual	Hypothesis
	AAGGGCCACGGA**T**TACTCGTAATAA		
	hypothesis:		
	AAGGGCCACGGA**G**TACTCGTAATAA		
Probes	——————————**A**——————————	PM	MM
	——————————**G**——————————	MM	MM
	——————————**T**——————————	MM	MM
	——————————**C**——————————	MM	PM
	01————————13————————25		

The top part of the graph shows two target sequences. The first sequence is the one that was used in the experiment that generates the data shown in Figure 1. The second sequence is a (wrong) hypothesis about sequence composition. These two sequences differ by a single nucleotide at base position 13. This base position is indicated by the numbers shown in the bottom. The microarray probes are grouped into four sets according to the type of nucleotide at the position where the two targets differ. The use of a wrong hypothesis leads to an error in the 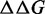 computation. For instance, probes with a nucleotide A at base position 13 are treated as having an AG mismatch (MM), while actually it has AT perfect match (PM).

In principle, for a unique sequence sample, one can start with any hypothesis and iterate until a collapsed 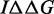 plot is found. However, when the sample is a mixture of two target sequences, the plots will always have branches, as over- and under-estimation of the data during the 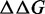 calculation will always occur. For each iteration, a new hypothesis is generated and when the 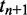 sequence is equal to the 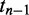 sequence as evaluated in block (d), the algorithm is stopped and gives as output a mixed sample composed by sequences *t_n_* and 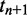.

## RESULTS AND DISCUSSION

### Decoding a set of coded samples

The algorithm was tested on seven coded clinical samples selected by Janssen Diagnostics out of a repository of samples for which the Sanger sequencing had been performed. The sequences composing these samples are shown in Table 3 where the left part of the Table shows the Sanger sequencing data provided as reference and the right part of the Table shows the sequences that were found by the algorithm. It can be seen that the proposed method decoded the samples successfully. To illustrate the working of algorithm, we discuss here in more details the sequences no. 3 and no. 4 of the seven sequences shown in Table 3, where the algorithm converges to a mixed and unique sequence, respectively. For convenience, in these two examples, the initial hypothesis *t*_1_ corresponds to the sequence with highest clinical frequency (ranked no.1 in Table 1). We tested that the algorithm also converges to the same results when *t*_1_ is one of the other 99 sequences of the PM set.

**Table 3.** Seven resequenced clinical samples from Janssen diagnostics

No.	Sanger sequencing	Proposed method
	179 180 181 182 183 184 185 186.	179 180 181 182 183 184 185 186.
1	GTT ATC TAT CAA TAC RTG GAT GAY T	GTT ATC TAT CAA TAC GTG GAT GAT T
		GTT ATC TAT CAA TAC ATG GAT GAC T
2	GTT ATC TGT CAA TAC ATG GAT GAT T	GTT ATC TGT CAA TAC ATG GAT GAT T
3	GTT ATC TAT CAR TAC ATG GAT GAY T	GTT ATC TAT CAA TAC ATG GAT GAC T
		GTT ATC TAT CAG TAC ATG GAT GAT T
4	GTT ATC TAT CAG TAC GTG GAT GAT T	GTT ATC TAT CAG TAC GTG GAT GAT T
5	GTT ATC TAT CAA TAC RTR GAT GAT T	GTT ATC TAT CAA TAC GTG GAT GAT T
		GTT ATC TAT CAA TAC ATA GAT GAT T
6	GTT ATC TAT CAA TAC RTG GAT GAC T	GTT ATC TAT CAA TAC ATG GAT GAC T
		GTT ATC TAT CAA TAC GTG GAT GAC T
7	GTT ATY TAT CAA TAC RTG GAT GAT T	GTT ATC TAT CAA TAC GTG GAT GAT T
		GTT ATT TAT CAA TAC ATG GAT GAT T

The numbers in the top row are indicating codon locations. Sequences on the left are the Sanger data whereas sequences on the right are the result from the method proposed in this article. The underlined nucleotides are mismatches against the sequence of highest clinical frequency including the degenerate bases i.e. R (purines: G or A) and Y (pyrimidines: C or T) (25). All sequences in this table are written in 5′ to 3′ orientation.

**Figure 4.**
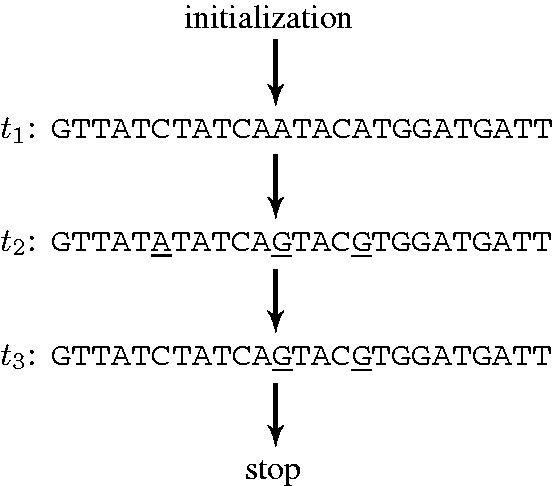
The hypothesis sequences from each iteration of the algorithm in the case of sample no. 4. It presents a conclusion that the sample contains a unique sequence *t*_3_ after three iterations.

The first example is sample no. 4. The left pane of Figure 3a shows an 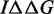 plot produced from the initial hypothesis *t*_1_; in this first iteration the data are very scattered. First, a set of data points (encircled) is selected. These are points that deviate the most from the expected thermodynamic behavior (dashed line). The nucleotide composition of this set is analysed: the right pane Figure 3a shows a histogram of mismatches of this selected set with respect to the initial hypothesis *t*_1_. In the algorithm, a threshold value of 70% is chosen as the minimum limit for the fraction of common mismatches per base positions between the selected probes and the current hypothesis (more details on the threshold selection and its influence on the output are presented in the Supplementary Section II). Based on this threshold, the selected probes are considered mismatching against the hypothesis *t*_1_ at base positions no. 6, 12 and 16, by nucleotides T, C and C, respectively. This information is used to generate a new hypothesis *t*_2_ by swapping nucleotides on hypothesis *t*_1_ so it becomes complementary to the three nucleotides detected in the mismatching base positions (resulting sequence *t*_2_ is given in Figure 4). The next iteration is the calculation of the 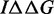 plot from *t*_2_, which is the graph shown in Figure 3b. Although, compared with *t*_1_, there is a better agreement with the expected thermodynamic behavior from Equation (1) (dashed line), the data are still scattered. The analysis of the most deviating set of points (encircled in Figure 3b) indicates that there is still a mismatch at base position no.6. The next *in silico* hypothesis *t*_3_ (Figure 4) is then obtained. The analysis of the most deviating set of points that corresponds to 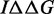 plot from *t*_3_ (Figure 3c) does not provide a strong signature for any common mismatch. Therefore, the algorithm concludes that sample no. 4 contains a unique sequence *t*_3_.

**Figure 5.**
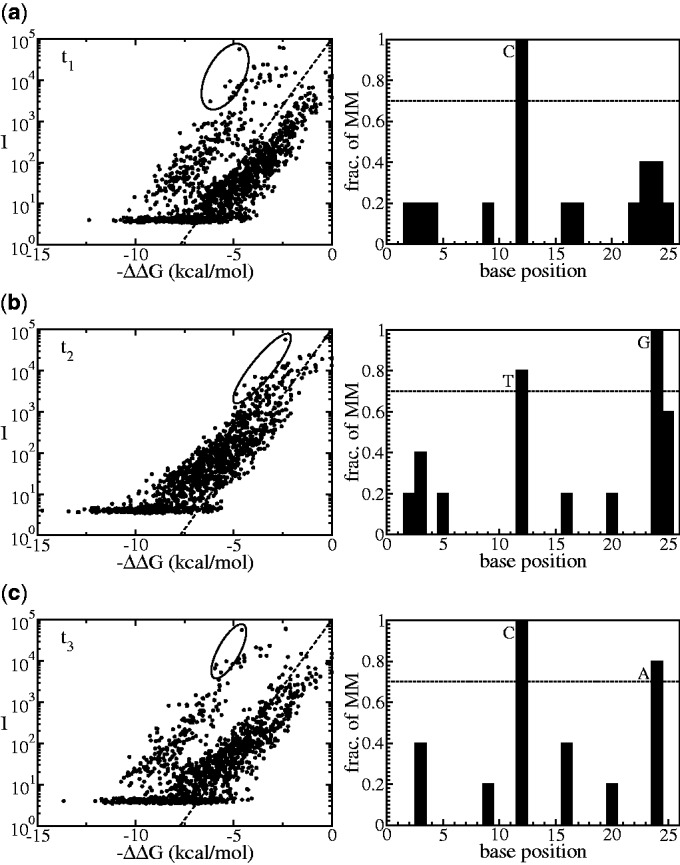
Analysis of sample no. 3, which is a mixed sample; in contrast to the analysis of unique samples such as shown in Figure 3, in the current case, the resulting 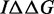 plots always have branches. Thus accordingly, fraction of mismatches higher than the threshold are always found in each iteration. In this Figure, results from analysing *t*_1_ (**a**), *t*_2_ (**b**) and *t*_3_ (**c**) are shown. Notice that the next hypothesis generated from *t*_3_, after implementing the nucleotide changes at position no. 12 and 24, is identical to *t*_2_. These sequences are shown in Figure 6. Therefore, the algorithm converges into two-cycle state and concludes that the sample in this current case is a mixed sequences sample.

**Figure 6.**
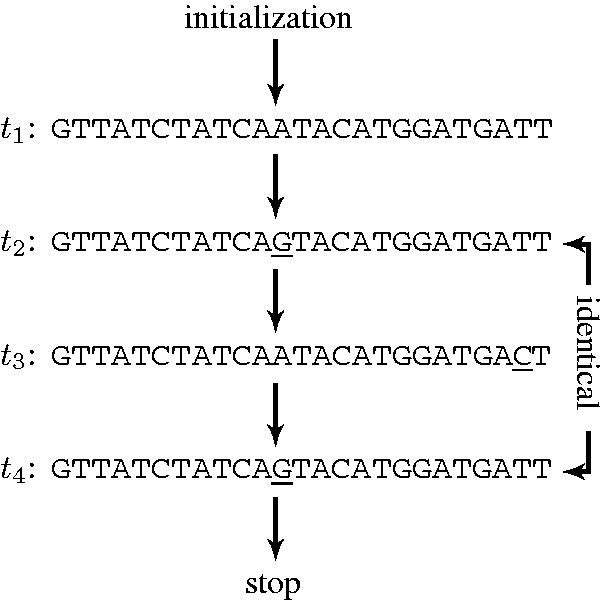
The hypothesis sequences from each iteration of the algorithm in the case of sample no. 3. It presents a conclusion that the sample contains mixed sequences as the generated hypothesis *t*_4_ after *t*_3_ is identical to previously generated hypothesis *t*_2_. This is a two-cycle state between 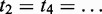 and 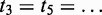.

The second example to be discussed is sample no. 3. Figure 5 shows the 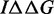 plots obtained during the iterations. In this case, the analysis of the most deviating branch of points generate a cycle in which sequences *t*_2_ and *t*_4_ are identical (Figures 6), indicating that the sample is actually a mixture of two sequences, differing in two nucleotides from each other. The difference with the case of sample no. 4 shown in Figure 3 is that in this case the 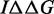 plot is always branched in all iterations forming a two-state cycle. The decision on whether a plot is branched or not is done by the algorithm based on the fraction of mismatches in the right panes, but note by visual inspection that in 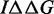 plot of hypothesis *t*_2_ (Figure 5b), the data points are quite close to the expected thermodynamic behavior of Equation (1) (dashed line). However, they are still more scattered compared with the case in Figure 3c, which is a unique sequence sample.

We comment on the differences between the quality of the collapses between Figure 1 and Figures 3 and 5. Figure 1 reports calibration experiments using a single synthetic target sequence and probes carrying up to two mismatches (15). In Figures 3 and 5, corresponding to hybridization experiments on HIV samples, the probes can carry more mismatches. The intensity drops to a constant background level for 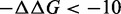 kcal/mol. Low intensity data have not been considered in the analysis as the algorithm selects the most deviating branch of points in a range 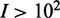. The HIV data are typically more scattered than those from the synthetic oligo, which is probably due to more involved sample preparation where there is still room for further improvement.

The microarray probes were designed to detect the top 100 sequences with highest clinical frequency, and mixtures thereof. The seven samples presented in this article all fall within this scope, and our algorithm produces the correct sample composition. A test based on our algorithm to reject sequences that are outside a diagnostic scope is further discussed in the Supplementary Section I.

### Resolving ambiguity from two degenerate bases

To discuss further about the result on mixed samples, we focused on degenerate nucleotides in the sequences due to uncertainties that are inherent in the Sanger method; as can be seen in the list of sequences on the left part of Table 3. The letters R and Y denote the degenerate bases A or G (purines) and C or T (pyrimidines), respectively (25). These uncertainties are caused by the presence of mixtures of two sequences in a given clinical sample. In the case of a single degenerate nucleotide, the sample is identified as a unique mixture. However, two different type of mixtures are possible in the case of two degenerate nucleotides in the same sequence. For instance a degenerate RR pair can mean either a AA/GG mixture or a AG/GA mixture. This type of ambiguity is present in the sequences no. 1, 3, 5 and 7 of Table 3. The analysis of the hybridization data from the microarray experiments allow to resolve this ambiguity because the hybridization free energies for each case are different.

The importance of this information lies in its clinical relevance, for example, in sample no. 5 where mutations RTR occur in codon 184. The presence of ATA as suggested by our method, gives an idea about the stage of resistance. This is interesting because during treatment with lamivudine, initially isoleucine mutants are present, which are subsequently replaced by valine variants (27). The isoleucine mutants are less fit than the valine mutants (28).

## CONCLUSIONS

In this article, we used inputs from hybridization thermodynamics to perform targeted resequencing of a fragment of the HIV-1 RT gene. In the HIV example considered here, due to the high mutation rate of the virus, the fragment analysed can occur in more than a hundred different variants. In addition, one needs to distinguish between samples composed by a single sequence (unique) from samples in which two or more different sequences coexist (mixed). For clinical purposes, it is important to identify early enough the rising of a resistant strain. Our microarray analysis is based on a large number of measured intensities not just from perfectly matching probes but also mismatching probes. Although in general the effect of the hybridization of a very low abundance sequence in a mixed sample can be small for each individual intensities, the correlated effect on a large number of different probes can be detected even from a target sequence at relatively low abundance. The analysis of (14), in which artificial synthetic mixtures of two sequences were used, indicated that the detection limit of relative abundance is of about 1%. In the Supplementary Section III, we repeat that analysis for the HIV data to illustrate the potential of the thermodynamic approach to microarray data analysis.

Here, we presented an iterative algorithm that successively generates *in silico* hypotheses for the sample composition and checked them against thermodynamic models. The algorithm identified correctly the sequences in the sample and was tested on seven clinical samples from the Janssen Diagnostics database. We showed how hybridization thermodynamics can resolve some intrinsic ambiguities of the Sanger sequencing. The results show the reliability of DNA microarrays and in principle any hybridization-based technology.

A method based on hybridization has the advantage that it is a simple test that can be miniaturized to a fully automated lab-on-chip, which can be used as point-of-care test. Indeed hybridization-based method is promising, and it has been a focus of interest in the recent years on either its fundamentals or applications (29–33). Although DNA microarrays are considered nowadays a mature technology and these devices are providing high quality and reproducibile data, their potentials have not been fully exploited. A better understanding of the underlying physico-chemical principles of hybridization is an issue of central interest and can lead to novel methods to improve the data analysis (34).

## SUPPLEMENTARY DATA

Supplementary Data are available at NAR Online, including [35].

## FUNDING

KULeuven [STRT1/09/042]; VITO [ZL39010200-401]. Funding for open access charge: VITO and Janssen Diagnostics.

*Conflict of interest statement.* None declared.

## Supplementary Material

Supplementary Data
