# Supplementary material for: Targeted resequencing of HIV variants by microarray thermodynamics
Source: Nucleic Acids Res. 2013 Aug 8;41(18):e173. doi: 10.1093/nar/gkt682 (PMC3794611; doi:10.1093/nar/gkt682)
Supplement: Supplementary Data [file supp_41_18_e173__index.html]

Targeted resequencing of HIV variants by microarray thermodynamics — Targeted resequencing of HIV variants by microarray thermodynamics — Supplementary Data 

# Targeted resequencing of HIV variants by microarray thermodynamics

## Supplementary Data

files

**Files in this Data Supplement:**

- Supplementary Data - pdf file
