# Supplementary material for: Targeted resequencing of HIV variants by microarray thermodynamics
Source: Nucleic Acids Res. 2013 Aug 8;41(18):e173. doi: 10.1093/nar/gkt682 (PMC3794611; doi:10.1093/nar/gkt682)
Supplement: Supplementary Data [file supp_gkt682_nar-00623-met-g-2013-File035.pdf]

Supplementary Data:
Targeted resequencing of HIV variants by microarray thermodynamics

W.W. Hadiwikarta,[1,2] B. Van Dorst,[3] K. Hollanders,[1] L. Stuyver,[3] E. Carlon,[2] and J. Hooyberghs[1,4]

[1] *Flemish Institute for Technological Research, VITO, Boeretang 200, B-2400 Mol, Belgium*
[2] *Institute for Theoretical Physics, KULeuven, Celestijnenlaan 200D, B-3001 Leuven, Belgium*
[3] *Janssen Diagnostics bvba, Turnhoutseweg 30, B-2340 Beerse, Belgium*
[4] *Theoretical Physics, Hasselt University, Campus Diepenbeek, Agoralaan - Building D, B-3590, Diepenbeek, Belgium*

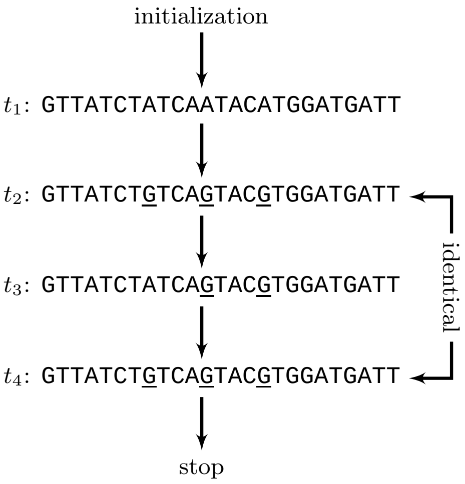

FIG. 1. The hypothesis sequences from iteration of the algorithm on sample no.4 with reduced probeset. Originally, this sample was found to be a unique sample, however after using the reduced probeset, the algorithm produced a cycle between two sequences. As summarized in Table I and discussed in the text, these sequences are outside of the scope of the reduced probeset and rejected.

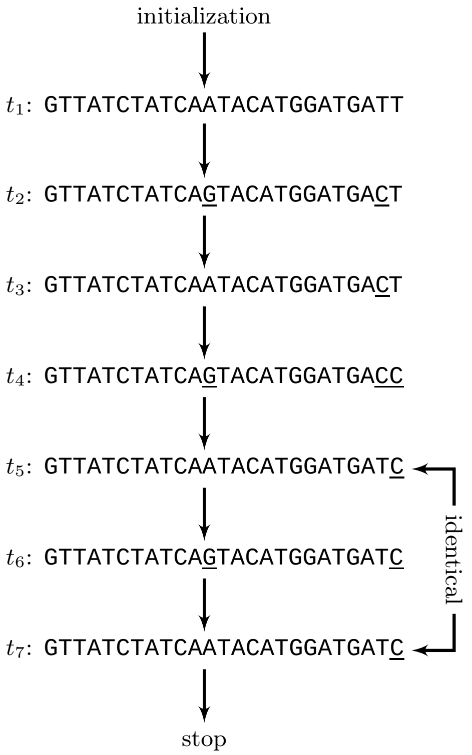

FIG. 2. The hypothesis sequences from each iteration of the algorithm on sample no.3 where we use the reduced probeset end up with a cycle between two sequences. These sequences are found to be outside of the scope as summarized in Table I and discussed in the text.

The aim of this text is to present some more details of the analysis of the data. Section I discusses a rejection test for samples outside the diagnostic scope of the design performed by running the algorithm on a reduced set of data. Section II discusses the sensitivity analysis to the threshold used to determine whether a $I\Delta\Delta G$ plot is collapsed or not. In Section III an analysis of a competive model of hybridization aimed at detecting low abundant targets is presented.

I. OUT-OF-SCOPE REJECTION TEST

The microarray design aims at detecting 100 sequences with the highest clinical frequencies and mixtures thereof. The algorithm was develop specifically to meet this aim. However, it should also be able to detect whether the sample analyzed contains sequences which are outside of the 100 most frequent ones, and reject them as they fall outside of this diagnostic scope. To test whether this actually happens in our algorithm, we have performed a *rejection test* for all the seven HIV samples. The algorithm was run for each sample on a reduced set of microarray data. To construct this reduced set, input from the Sanger sequencing was used as follows. Suppose that a sample contains a pure sequence t_i and let us indicate with p_i, its perfectly matching probe in the microarray. We then discarded from the data analysis, the probe p_i and those with one or two nucleotides differing from p_i. By restricting the set of analyzed data, we induce each sample to fall outside the diagnostic scope. The diagnostic scope now contains typically less than 99 sequences as discarding probes with one or two nucleotide difference with respect to p_i, might remove other sequences in the original diagnostic set of 100. Let us indicate this reduced diagnostic set with S_i. An important test of the

Sample Nr.	Correct sequence(s) detected with original probeset	Sequence(s) detected with reduced probeset	Status
4	GTTATCTATCAGTACGTGGATGATT	GTTATCTATCAGTACGTGGATGATT	*Outside* → *Rejected*
		GTTATCTGTCAGTACGTGGATGATT	*Outside* → *Rejected*
3	GTTATCTATCAATACATGGATGACT	GTTATCTATCAATACATGGATGATC	*Outside* → *Rejected*
	GTTATCTATCAGTACATGGATGATT	GTTATCTATCAGTACATGGATGATC	*Outside* → *Rejected*

TABLE I. Results from applying the algorithm on samples with reduced probesets and comparing them to results from previous analysis with original probeset as shown in the main article. As can be seen in column 3, the outcome are sequences that are either perfectly matching or having one or two mismatches against the correct sequences shown in column 2. Therefore, they are associated to the removed probes and regarded as outside of the scope and rejected.

robustness of our analysis is that the algorithm should not converge to any of the sequences in S_i. In the case that the original sample is mixed, the reduced set was obtained by removing the union of probes associated to either target sequence comprising the mixture.

Figures 1 and 2 illustrate two examples in which the algorithm was run on a reduced set of data. The two samples are those discussed in the main article (sample no.4 and 3 respectively from the Table 3 of the article). In both cases, the starting hypothesis t_1 is the sequence of highest clinical frequency. Table I summarizes the results and shows a comparison to the sequences obtained from the analysis of the full microarray data. For the sample no.4, while the original analysis indicates it as a unique sample, the run on a reduced set of probes suggests that the sample is composed by a mixture of two sequences. We note that one of the sequences in this mixture is identical to the correct one, while the second one contains an additional mismatch with a G replacing an A. However, both detected sequences fall outside the diagnostic set S_i and are consequently rejected by the algorithm. A similar result is obtained from the analysis of the sample no.3. Here the run on the reduced set indicates that the target composition is a mixture of two sequences. The analysis fails to identify correctly the nucleotides close to the double helix edge. Also in this case the identified sequences fall outside the diagnostic set S_i and are thus rejected.

As a conclusion, the diagnostic test aims at identifying the presence of some predefined sequences (the diagnostic set) and mixtures thereof. By restricting the analysis to a subset of microarray data we have shown that the algorithm correctly detects whether the sequences obtained from the analysis fall outside the diagnostic set.

II. $I\Delta\Delta G$ COLLAPSE ANALYSIS: SENSITIVITY TO THRESHOLD VALUES

One of the crucial points of the algorithm for the target identification is the decision whether an $I\Delta\Delta G$ plot is collapsed (*unique sample*) or branched (*mixed sample*). This decision is based on the analysis of the most deviating branch of data points and uses a threshold parameter. The threshold sets the minimum fraction of common mismatching nucleotides in the deviating branch to identify a novel sequence hypothesis in comparison to the hypothetical sequence of the prior iteration. This threshold is set in the program as 0.70 (see main article, horizontal lines in right panes of Figure 3 and 5). This value was chosen as a compromise between high threshold values which usually oversimplify the sample composition (some mismatches can be missed), and low threshold values which may overestimate the complexity of the sample. Our analysis was repeated for different threshold values and we found that the results are independent on the threshold value if this is chosen in the range of $0.60 <$ threshold < 0.80. Thus the chosen value of 0.70 is within this interval. To illustrate this, Table II shows the sample composition (for the sample no.3 of the main article) obtained from the algorithm for different threshold values; the first column gives the threshold values and the second column shows the detected sequences. The third column gives the sample composition in Sanger notation (R means A or G, Y means T or C, etc.). In this example changing the threshold affects the nucleotides close to the border, as shown when the threshold is too high a mismatch close to the border is missing and when the threshold is too low an extra mismatch is added (thus increases the complexity of the sample).

III. LOW ABUNDANT TARGET IDENTIFICATION

Due to the very high mutation rate of the HIV-1, one may wonder if it is possible to detect sequences in minority that coexist with one unique majority sequence in the same sample. In here, we explore further the hybridization thermodynamics to address this issue. Even if the algorithm converges to a unique sequence, the data can be further analyzed to look for the signature of a second low abundance sequence. In a general case where the sample contains a mixture of two sequences i.e. t_a and t_b, one expects that the measured intensity follows a competitive hybridization model:

$$I_m(c_a, c_b) = A\left[c_a e^{-\Delta G_a/RT} + c_b e^{-\Delta G_b/RT}\right] \quad (1)$$

where A is again a proportionality factor, c_a and c_b are the respective concentrations of the two sequences

Threshold	Sequences detected	Sanger notation
0.90	GTTATCTATCAATACATGGATGACT<br>GTTATCTATCAGTACATGGATGACT	GTTATCTATCARTACATGGATGACT
0.85	GTTATCTATCAATACATGGATGACT<br>GTTATCTATCAGTACATGGATGACT	GTTATCTATCARTACATGGATGACT
0.80	GTTATCTATCAATACATGGATGACT<br>GTTATCTATCAGTACATGGATGACT	GTTATCTATCARTACATGGATGACT
0.75	GTTATCTATCAATACATGGATGACT<br>GTTATCTATCAGTACATGGATGATT	GTTATCTATCARTACATGGATGAYT
0.70	GTTATCTATCAATACATGGATGACT<br>GTTATCTATCAGTACATGGATGATT	GTTATCTATCARTACATGGATGAYT
0.65	GTTATCTATCAATACATGGATGACT<br>GTTATCTATCAGTACATGGATGATT	GTTATCTATCARTACATGGATGAYT
0.60	GTTATCTATCAATACATGGATGACT<br>GTTATCTATCAGTACATGGATGATT	GTTATCTATCARTACATGGATGAYT
0.55	GTTATCTATCAATACATGGATGACC<br>GTTATCTATCAGTACATGGATGATT	GTTATCTATCARTACATGGATGAYY
0.50	GTTATCTATCAATACATGGATGACC<br>GTTATCTATCAGTACATGGATGATT	GTTATCTATCARTACATGGATGAYY

TABLE II. An example of the impact on using different thresholds in finding the sequence composition. In this case sample no.3 from Table 3 of the main article is used. The correct sequence composition is independent to the threshold provided it is in the range of $0.60 < \text{threshold} < 0.80$. As the threshold goes high $(0.80 - 0.90)$, the algorithm missed a mismatch (at base position 24) and if the threshold is lowered $(0.50 - 0.55)$, an extra mismatch is added (at base position 25).

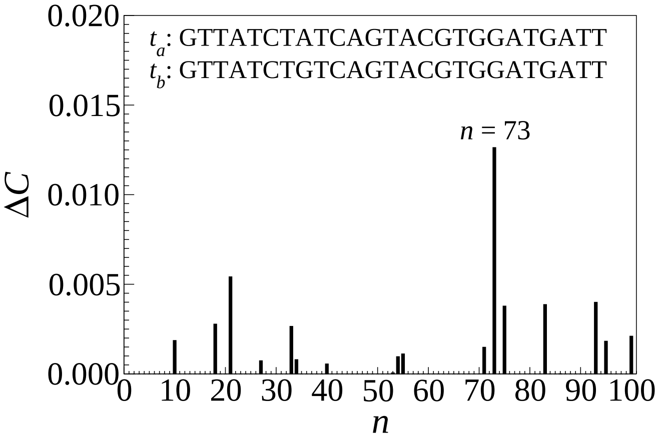

FIG. 3. The result of calculating ΔC following Equation (2), for each sequence in the PM set using hybridization data of sample no.4 from Table 3 of the main article. From the calculation, it turns out that sequence $n = 73$ is giving the highest value of ΔC thus considered as minority in the sample. The sequences t_a and t_b are the majority sequence and minority sequences respectively.

composed the mixed sample, ΔG_a and ΔG_b are the hybridization free energies between the probe sequence and target sequences t_a and t_b respectively. Equation (1) in this supplementary material generalizes Equation (1) of the main article in the case of a mixture under the same thermodynamics assumptions. Having identified the majority sequence e.g. sequence t_a by using the algorithm discussed previously, we use the competitive hybridization model (Equation (1)) to search for a possible minority sequence t_b. This is done by maximizing the increase of the correlation between experimental intensities data I and the model for these intensities $I_m(c_a, c_b)$ i.e. Equation (1). For a given sequence t_b we can write this maximization as

$$\Delta C\,(t_b) \equiv \max_{c_b}(corr(I, I_m(c_a, c_b))) - corr(I, I_m(c_a, 0)). \tag{2}$$

The first term in Equation (2) is the maximum correlation between I and $I_m(c_a, c_b)$ that can be achieved by gradually increase c_b in the computation. Therefore, this first term determines the relative concentration c_b/c_a in the sample. The second term is the correlation between experimental intensities I and $I_m(c_a, c_b)$ if there is no second sequence exists in the sample ($c_b = 0$). Therefore, we are looking for a specific t_b that gives the highest ΔC.

Figure 3 shows a plot of ΔC as a function of the sequence t_b from the list of the top 100 unique sequences used in the test (PM set), indexed by notation $n = 1, \ldots, 100$. We use hybridization data from sample no.4 of Table 3 from the main article, in which the described algorithm converged to a unique sequence sample. As can be seen in this Figure 3, most target sequence from the PM set gives $\Delta C = 0$, implying that introducing a second sequence decreases the correlation between Equation 1 and the intensities data, hence the maximum in ΔC is obtained from the case $c_b = 0$. Few cases however, show an increase in correlation, partic-

		sequence	relative clinical
rank	type	...180...182...184...186.	frequency
1	unique	GTTATCTATCAATACATGGATGATT	1.0000
2	unique	GTTATCTATCAATAC**G**TGGATGATT	0.4115
3	unique	GTTATCTATCAATACATGGATGA**C**T	0.1239
4	unique	GT**C**ATCTATCAATACATGGATGATT	0.0845
5	unique	GTTATCT**G**TCAATACATGGATGATT	0.0752
		⋮	
9	unique	GTTATCTATCAATAC**G**TGGATGA**C**T	0.0560
		⋮	
15	mixed	GTTATCTATCAATAC**R**TGGATGATT	0.0373
		⋮	
22	unique	GT**C**ATCTATCAATA**T**ATGGATGA**C**T	0.0266
		⋮	
143	unique	GTTATCTATCAATACATGGATGA**CC**	0.0022
		⋮	
319	mixed	GTTATCT**R**TCAATACATGGATGATT	0.0004
		⋮	

TABLE III. This Table is from the main article. We show it again in this Supplementary Material to indicate the sequence observed by the correlation analysis. Nucleotide sequences (from codon 179 to codon 186, written in 5' to 3' orientation) for different variants of the HIV-RT gene, as obtained from the analysis of 350,000 patients. The database, provided by Janssen Diagnostics, is obtained from Sanger sequencing, and only some selected sequences are shown here. The ranking follows the relative clinical frequency. Numbers above the first ranked sequence indicate the codons position. Nucleotides differing from those of the most frequent sequence are shown in bold. The Sanger sequencing method yields either *unique* sequences, i.e. with no ambiguities, or *mixed* sequences. In the Table, the mixed sequence with the highest clinical frequency is ranked no.15. We use here the standard notation for degenerate bases, therefore R means a purine (A or G). Note that this sequence is a mix between sequences ranked no.1 and no.2 in the Table. The sequence ranked no.143 is the 100th unique sequence from the database and so is the last in the PM set. Sequence rank no.319 is the mixed sample that is observed by the correlation analysis discussed in Section III.

ularly sequence $n = 73$ of this PM set. The sequence corresponds to $n = 73$ is shown in Figure 3 as t_b, while t_a is the sequence in majority. These sequences differ by a single nucleotide, where an A is replaced by a G at base position no.8. We recall that the mutation from A to G is a common one in HIV [1], which makes the presence of sequence no.73 biologically very plausible. The combination of sequences obtained from the correlation analysis is also observed in the Janssen Diagnostics Sanger database; this is the entry no.319 shown in Table III. Thus it is suggested that what the Sanger sequencing identified as a unique sequence (entry no.4 in Table 3 of the main article) is actually a mixed sequence sample, where the concentration of the sequence in minority would be below the threshold of detection of the Sanger method.

We note indeed that the increase of correlation shown in Figure 3 is small. However, it occurs in a significant way only for one sequence out of the 100 analysed. From Equation 1 one can also estimate the relative abundance of the mixture; we found $c_b/c_a \approx 0.01$, so the minority sequence is present at 1%. A previous publication [2] dedicated to the analysis of mixtures of synthetic oligomers with different relative abundance estimated the detection limit of the method to be of about 1%.

[1] Mullins, J. I., Heath, L., Hughes, J. P., Kicha, J., Styrchak, S., Wong, K. G., Rao, U., Hansen, A., Harris, K. S., Laurent, J.-P., Li, D., Simpson, J. H., Essigmann, J. M., Loeb, L. A. and Parkins, J. (2011) Mutation of HIV-1 Genomes in a Clinical Population Treated with the Mutagenic Nucleoside KP1461. *PLoS ONE*, **6**, e15135–.

[2] Hooyberghs,J. and Carlon,E. (2010) Hybridisation thermodynamic parameters allow accurate detection of point mutations with DNA microarrays. *Biosens. Bioelectron.*, **26**, 1692–1695.
